# Trends in cancer incidence and mortality in the process of metropolitanization of Shanghai, 1973–2017

**DOI:** 10.3389/fonc.2025.1615492

**Published:** 2025-08-01

**Authors:** Jiejie Qin, Mengyin Wu, Shulin Zhao, Kai Gu, Renzhi Cai, Ziwei Tang, Defeng Zhu, Jingyan Tian, Wei Yao, Baiyong Shen, Yan Shi

**Affiliations:** ^1^ Department of General Surgery, Pancreatic Disease Center, Ruijin Hospital, Shanghai Jiao Tong University School of Medicine, Shanghai, China; ^2^ Shanghai Key Laboratory of Pancreatic Neoplasms Translational Medicine, Shanghai Jiao Tong University School of Medicine, Shanghai, China; ^3^ Research Institute of Pancreatic Diseases, Shanghai Jiao Tong University School of Medicine, Shanghai, China; ^4^ Clinical Research Center, Ruijin Hospital, Shanghai Jiao Tong University School of Medicine, Shanghai, China; ^5^ Division of Noncommunicable Diseases and Injury, Shanghai Municipal Center for Disease Control and Prevention, Shanghai, China; ^6^ Department of Vital Statistics, Division of Health Information, Shanghai Municipal Center for Disease Control and Prevention, Shanghai, China; ^7^ Shanghai Institute of Microsystem and information Technology, Chinese Academy of Sciences, Shanghai, China; ^8^ Shanghai Institute of Endocrine and Metabolic Diseases, Department of Endocrinology and Metabolism, Ruijin Hospital, Shanghai Jiao Tong University School of Medicine, Shanghai, China

**Keywords:** cancer, incidence, mortality, trend, socioenvironment, metropolitanization

## Abstract

**Background:**

Shanghai has become a modern and international metropolis. A more comprehensive understanding of cancer incidence and mortality rates and socioenvironmental factors is explored to develop effective cancer control policies in Shanghai.

**Methods:**

Cancer registration data are currently collected in Shanghai from 1973 to 2017, and socioenvironmental factors were obtained from the Shanghai statistical yearbook. Multivariate ridge regression analysis explored the contributions of socioenvironmental factors to cancer incidence and mortality, and the estimated annual percentage change (EAPC) was calculated for each cancer type by gender and district.

**Results:**

Multivariate ridge regression analysis indicated that the number of divorces, total waste gas from industry, areas of buildings completed, and number of computers probably drove the increase in cancer incidence, and health expenditure and medical insurance cost probably contributed to the decrease in cancer mortality in Shanghai. Age-standardized cancer incidences of the lung in female patients, prostate, thyroid, and cervix increased most, and the incidence and mortality of esophagus, liver, and stomach cancers decreased most in Shanghai from 2002 to 2017. The most common cancer sites diagnosed were lung, colorectal, female breast, and male prostate in Shanghai in 2017, similar to the pattern in high-income countries. Stricter air control strategies, lower divorce rates, healthier lifestyles, and more effective HPV vaccination campaigns may be useful actionable measures of cancer prevention.

**Conclusions:**

The longitudinal cancer data from the real world, which span decades, reported here and Shanghai’s experience in cancer prevention and control can be a reference for government guidelines in preventing population-level cancer incidence during city development.

## Introduction

Cancer has been the leading cause of death and a significant public health issue in China (1). Shanghai is a significant economic and cultural center in China, and its rapid metropolitanization may cause population growth or aging or sociodemographic changes (2), which may impact cancer burden. Earlier studies have documented cancer burden in Shanghai, highlighting trends in cancer incidence in urban Shanghai from 1973 to 2010 (3) and urban–rural disparities in cancer incidence and mortality from 2002 to 2015 (4). However, it is rarely known whether socioenvironmental factors impact cancer incidence and cancer mortality in the process of the metropolitanization of Shanghai.

A more comprehensive understanding of cancer incidence and mortality and socioenvironmental factors is crucial to formulate effective cancer control policies. Therefore, this study presented cancer incidence and mortality trends in Shanghai, potential socioenvironmental factors probably attributing to these trends, cancer incidence and mortality pattern, and their implications for cancer control in Shanghai.

## Methods

### Data source

To comprehensively investigate and interpret the change of cancer trends, cancer registration data are currently collected in urban Shanghai from 1973 to 2017 and in rural Shanghai from 2002 to 2017. Factors associated with society and environment were collected from the Shanghai statistical yearbook, including population, population density, GDP per capita, total waste gas from industry, computers per hundred families, areas of buildings completed, divorce, total health expenditure, and medical insurance cost. Cancer registration utilizes internationally recognized ICD-10 codes for classification. Single cancer site and site groups comprising multiple sites were analyzed as the predominant causes of cancer incidence and death. Cancer sites in this study included nasopharynx (C11), esophagus (C15), stomach (C16), bowel (C18-20), liver (C22), gallbladder (C23-24), pancreas (C25), lung (C33-34), breast (C50), cervix (C53), uterus (C54), ovary (C56), prostate (C61), kidney (C64), bladder (C67), brain and central nervous system (C70-72), thyroid (C73), lymphoma (C81-86, C96), and leukemia (C91-C95). This study has been reviewed and approved by the Ethics Committee of Shanghai Municipal Center for Disease Control and Prevention.

### Statistical analysis

EAPC values were calculated using a reported method of a generalized linear regression model with quasi-Poisson link function (5, 6). The study measured age-standardized cancer incidence and mortality rates per 100,000 as the outcome, with time from 2002 to 2017 serving as the independent variable. EAPC was estimated using the formula (exp (β^−1)’ 100, where β^ represented the estimated slope of the period variable. The 95% confidence interval (CI) is calculated from the fitted quasi-poison regression model (7). Trend analysis was based on the methodology outlined in the published paper (5). In brief, the criteria were followed: firstly, an increasing trend was identified when the EAPC value exceeds zero and the corresponding *p*-value was less than 0.05, indicating a notable upward trend with statistical significance. Secondly, a statistically significant decline trend was observed when the EAPC value was below 0 and the corresponding *p*-value was less than 0.05. Ultimately, when these conditions were unmet, cancer trends were determined to have remained stable. Univariate linear regression and multivariate ridge regression are used to quantify the contribution of each factor on cancer incidence and mortality trends. R version 4.4.2 was used for statistical analysis (8).

## Results

### Socioenvironmental factors could influence cancer incidence and mortality trends in Shanghai

**Figure 1:**
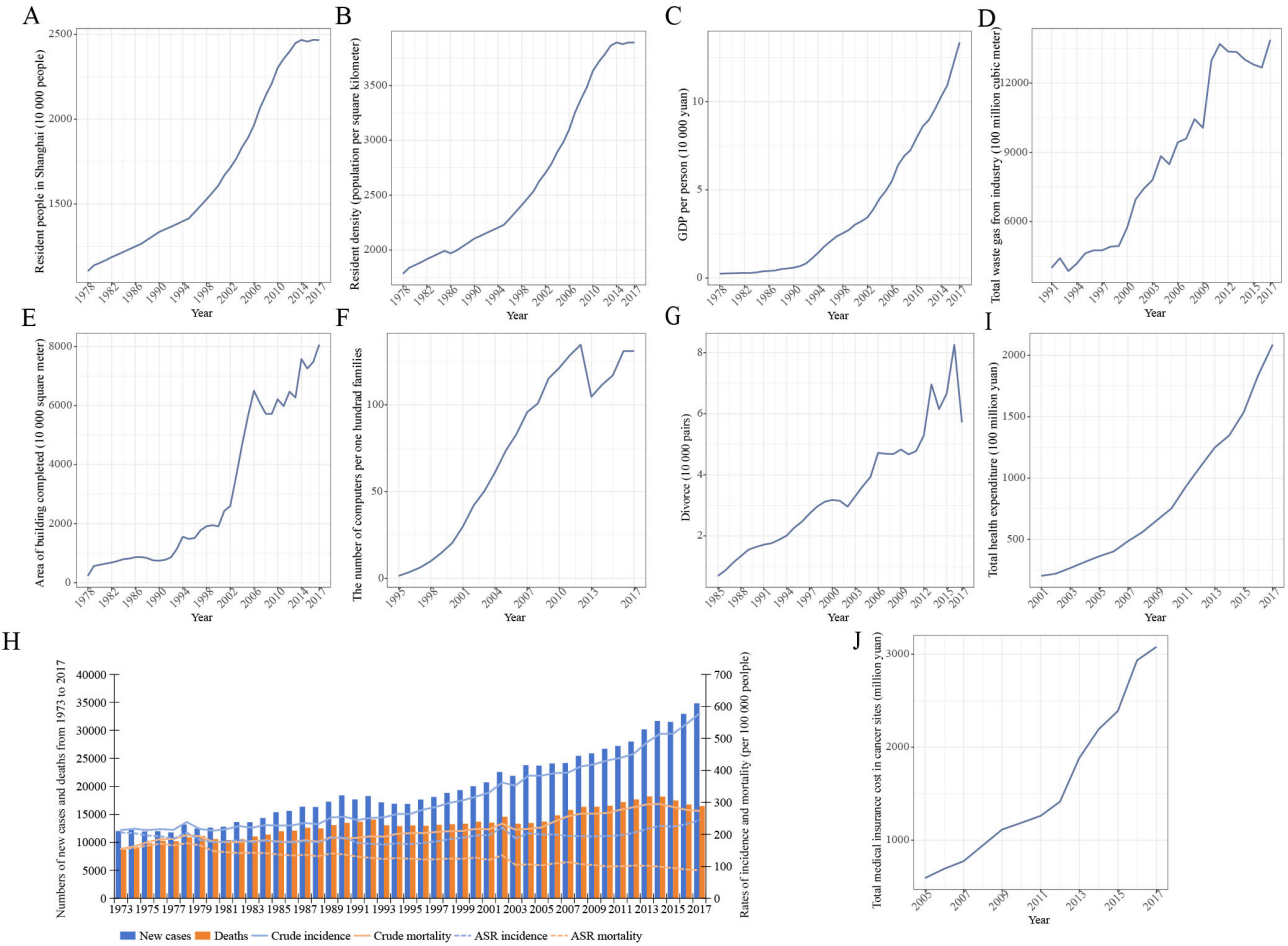
Analysis of socioenvironmental trends alongside cancer incidence and mortality rates over time in Shanghai. **(A–C)** Trends in population, population density, and GDP per capita in Shanghai from 1978 to 2017. **(D–G)** Trends in total waste gas from industry, area of building completed, the number of computers per hundred families, and the number of divorces over time in Shanghai. **(H)** Analysis of crude and age-standardized cancer incidence and mortality in urban Shanghai from 1973 to 2017. **(I, J)** Trends in total health expenditure and medical insurance cost for all cancers over time in Shanghai.

Between 1978 and 2017, Shanghai transformed into a modern international metropolis, experiencing significant growth in population, population density, and GDP per capita (**Figures 1A–C**). The economic growth and increasingly urbanized and westernized lifestyle resulted in increasing environmental pollution (9). During the metropolitanization of Shanghai, outdoor and indoor environmental pollution (increases in waste gas from industry and buildings completed, **Figures 1D, E**), westernized lifestyles reflected by the increasing number of computers (**Figure 1F**), and mental stress (more divorce cases and higher price of real estate, **Figure 1G**)—all of these socioenvironmental factors were positively correlated with cancer incidence (**Table 1**), probably driving the increase in new cancer cases by 189.9% from 12,008 in 1973 to 34,810 in 2017 and an increase in crude cancer incidence by 170.6% from 213.4 in 1973 to 577.5 per 100–000 in 2017 in urban Shanghai, respectively (**Figure 1H**). Even though we adjusted the impact of age using Segi’s world population in 1960, an increase in age-standardized cancer incidence was observed in urban Shanghai from 1973 to 2017. In addition, screening projects and advanced early-stage diagnosis technology may contribute to the upward trend in cancer incidence. Facing the severe cancer burden, Shanghai made more efforts to improve the prognosis of cancer patients. Increasing health expenditure and medical insurance cost were negatively correlated with cancer mortality (**Figures 1I, J**, **Table 1**), probably attributing to the decline of age-standardized cancer mortality in urban Shanghai between 1973 and 2017 (**Figure 1H**). The increasing crude mortality may be the result of aging and population increase over time (**Figure 1H**).

**Table 1:** Correlation analysis between cancer incidence and mortality and socioenvironmental factors.

Dependent variable	Independent variable	Univariate linear regression analysis		Multivariate ridge regression
		Standard coefficient	*P*-value	Standard coefficient
Incidence	Divorces	0.915	<0.0001	0.314
	Waste gas from industry	0.702	<0.0001	0.303
	Area of buildings completed	0.47	<0.0001	0.224
	Number of computers per 100 families	0.381	<0.0001	0.083
Mortality	Health expenditure	-0.109	<0.0001	-0.546
	Medical insurance cost for cancers	-0.112	<0.0001	-0.064

### Individual cancer incidence and mortality trends during Shanghai’s metropolitanization

**Figure 2:**
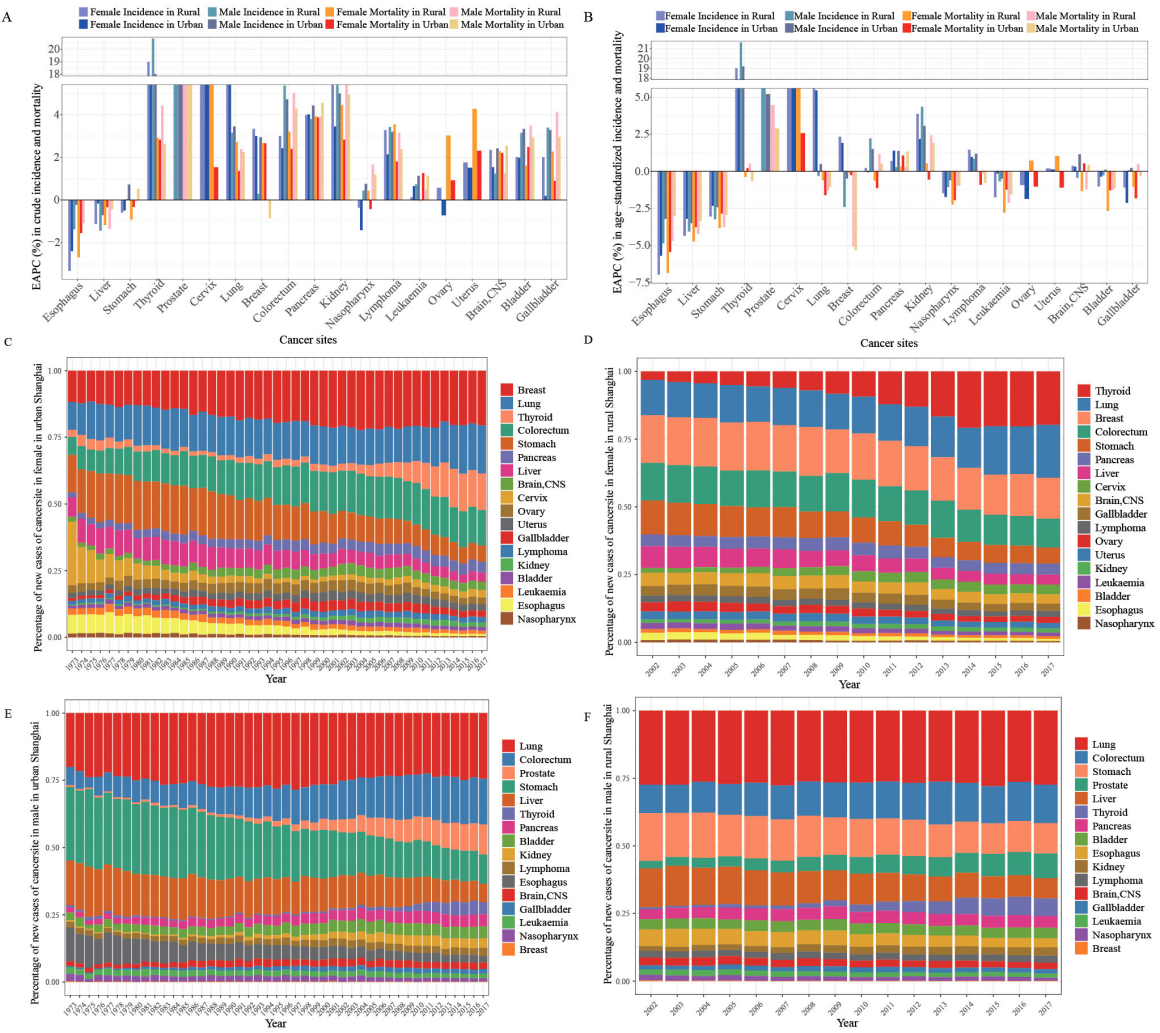
Incidence and mortality trends of each cancer type and cancer patterns in Shanghai, 2002–2017. **(A, B)** Estimated annual percentage change (EAPC) of crude and age-standardized cancer incidence and mortality categorized by gender and district in Shanghai from 2002 to 2017. **(C, D)** Stacked bar plots depict the cancer patterns of new cases in women in urban, 1973–2017, and in rural, 2002–2017. **(E, F)** Stacked bar plots depict the cancer patterns of new cases in men in urban, 1973–2017, and in rural, 2002–2017.

We further calculated the EAPC for each cancer type by gender and district from 2002 to 2017. The EAPCs based on crude rates for most cancer sites except liver, esophagus, and stomach are more than 0, indicating upward trends both in crude cancer incidence and cancer mortality from 2002 to 2017 (**Figure 2A**). However, the EAPC values based on age-standardized cancer incidence and mortality for most cancer sites (esophagus, liver, stomach, etc.) sharply decreased, especially for cancer mortality (**Figure 2B**). Population growth and aging probably led to the opposite results.

**Figure 3:**
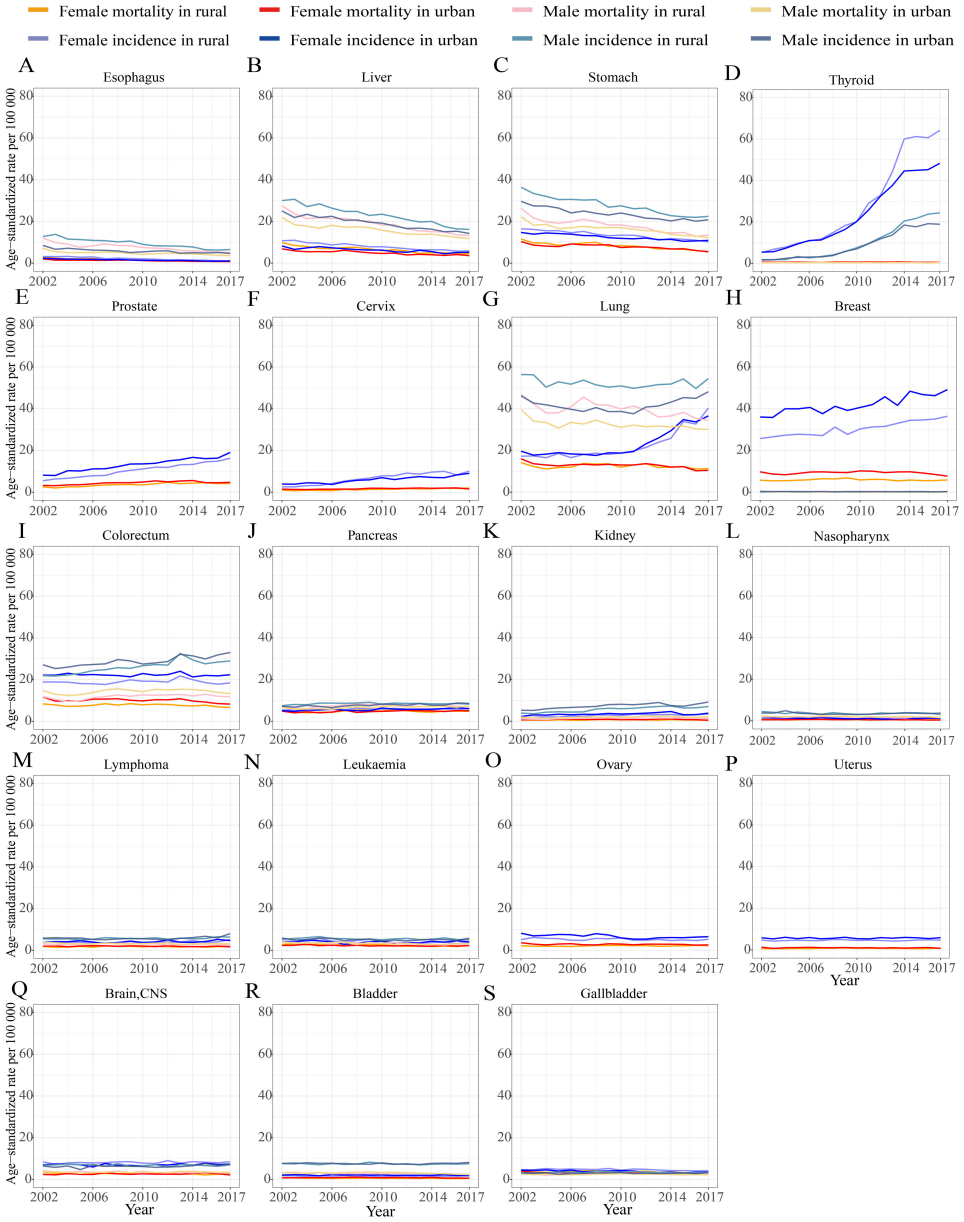
Trends in individual cancer incidence and mortality standardized by Segi’s world population in 1960, categorized by gender and district in Shanghai from 2002 to 2017. **(A)** esophagus; **(B)** liver; **(C)** stomach; **(D)** thyroid; **(E)** prostate; **(F)** cervix; **(G)** lung; **(H)** breast; **(I)** colorectum; **(J)** pancreas; **(K)** kidney; **(L)** nasopharynx; **(M)** lymphoma; **(N)** leukaemia; **(O)** ovary; **(P)** uterus; **(Q)** brain and central nervous system (brain, CNS); **(R)** bladder; **(S)** gallbladder.

The incidence and mortality trends of specific cancer sites differed by gender and district over time (**Figures 2B**, **3**). The incidence and mortality of esophagus, liver, and stomach cancers decreased by more than 2% per year both in rural and urban locations from 2002 to 2017. The top-four increase in age-standardized incidence was observed in cancers of the lung in females, prostate, thyroid, and cervix, with more than 5.0% increase per year (**Figure 2B**). In contrast to the upward incidence trend, the mortality of thyroid cancer remained very low and stable at less than 1.5% (**Figure 2B**). However, trends in mortality of prostate and cervix cancer increased by more than 2% per year, especially in rural areas. Lung cancer incidence in female patients sharply increased at 5.4% and 6.0% per year in urban and rural areas, respectively, while incidences in male patients hardly remained stable from 2002 to 2017 (urban EAPC of 0.46%, 95% CI (-0.30, 0.23); rural EAPC of -0.29%, 95% CI (-0.71, 0.14)). The age-standardized mortality of lung cancer slightly decreased in all populations. The age-standardized incidences of breast and colorectal cancer rose over time. Female breast cancer incidence, both in urban and rural areas, increased by more than 1.5% per year, while mortality in both rural and urban areas remained stable. The incidence and mortality of male colorectal cancer increased, especially in rural incidence, while the female colorectal cancer rates remained stable. Pancreas and kidney cancer incidence and mortality rates all slightly increased in all population, except in female mortality in urban areas.

Furthermore, we made a sensitivity analysis to present the cancer trends in urban (1973–2017) and in rural (2002–2017) areas (**Supplementary Figures S1A, B**). The results showed similar trends except cancer sites in cervix and gallbladder. **Supplementary Figure S1C** shows that the upward trend in cervix cancer in urban areas from 2002 to 2017 is consistent with that in rural Shanghai from 2002 to 2017, while the downward trend in cervix cancer in urban areas from 1973 to 2002 resulted in the opposite trends in urban areas, between **Supplementary Figures S2A, B** and **Supplementary Figures S1A, B**, contributed by the widespread application of human papillomavirus (HPV) vaccines. Similarly, the upward trends in gallbladder cancer incidence and mortality in urban Shanghai in 1973 to 2002 are opposite to the downward trend in gallbladder cancer in rural and urban Shanghai in 2003–2017 (**Supplementary Figure S1D**).

### Cancer incidence and mortality patterns in Shanghai

During the metropolitanization of Shanghai, the cancer incidence profile seemed to be increasingly similar to the patterns of high-income countries (10), such as United States (11) and Japan (12). For women, the mostly frequently diagnosed cancer sites were cervix, stomach, and breast in urban Shanghai in 1973, which had been breast, colorectal, and lung cancers in urban and rural Shanghai since 2002, similar to the patterns of highly developed countries (**Figures 2C, D**). The proportion of new cases of breast cancer in urban Shanghai increased from 11.77% to 20.53% from 1973 to 2017, higher than 15.01% in rural areas in 2017. For men, the most frequently diagnosed cancer sites were lung, stomach, colorectal, and liver in rural and urban Shanghai in 2002. By 2017, the prevalent cancer sites had shifted to lung, colorectal, prostate, and stomach (**Figures 2E, F**). Specially the proportion of new cases of stomach cancer in male patients in urban Shanghai decreased from 27.31% to 10.91% from 1973 to 2017, only a little lower than 11.21% in men in rural Shanghai in 2017. The cancer types of lung, colorectal, male prostate, and female breast may be the universal cancer incidence patterns in metropolitanized cities. Westernized lifestyles, environmental pollution, effective prevention, and healthcare awareness promotion may be the reason for these pattern transformations in the process of metropolitanization.

However, the cancer mortality profiles did not seem to change from 2002 to 2017. For cancer deaths, lung, colorectal, stomach, liver, and pancreas had been the top five cancer sites in men, similar to that of lung, colorectal, stomach, breast, and pancreas in women in urban and rural Shanghai in 2017 (**Supplementary Figure S2**). The proportion of deaths due to colorectal cancer rose from 1973 to 2017 in urban Shanghai, which became the second leading cause of cancer deaths (**Supplementary Figures S2A, B**).

### Cancer prevention and control in Shanghai

Lung cancer and colorectal cancer were the top two causes of new cancer cases and cancer deaths in Shanghai in 2017, highlighting the need for more efforts on enhanced prevention and control strategies, such as stricter air quality controls and healthier lifestyles. In 2002, the Shanghai Municipal Government initiated a colorectal cancer screening program in communities. Great progress was archived on the initial screening completion, with colorectal cancer detection rates of 201.35/100,000 in 2013 (13). Next, screening programs for cancers of the lung, stomach, esophagus, liver, breast, and cervix have been initiated in Shanghai since 2013. In addition, “Healthy Shanghai 2030” and “Healthy Shanghai Action (2019–2030)” projects were initiated in 2019, both emphasizing more effective tobacco control strategies, healthy lifestyles, and control measures of environment pollution. Notably, lung and breast cancer incidences and mortality among women have increased significantly. The corresponding prevention and control measures should be taken, such as healthier lifestyles and reduction of indoor pollution and divorce rates. Even though the trend in cervix cancer incidence sharply dropped before 2002 due to the widespread uptake of HPV vaccines, the upward trend since 2002 indicated the need for more effective HPV vaccination campaigns.

We believe that the longitudinal cancer data from the real world, which span decades, reported here and Shanghai’s experience in cancer prevention and control can be a reference for government guidelines in preventing population-level cancer incidence during city development.

## Discussion

During the recent decades, the rapid metropolitanization of Shanghai brought dramatic changes due to socioenvironmental factors (14). A multivariate ridge regression analysis indicated that some socioenvironmental factors probably contributed to cancer incidence, including number of divorces, total waste gas from industry, buildings completed, and number of computers per 100 families (minimum contribution). The dramatic increase in total waste gas from industry and the areas of building completed may increase the outdoor air pollution, especially particulars (PM_2.5_), and indoor air pollution (formaldehyde and benzene), respectively, which are attributed to lung cancer incidence (15) and cancer burden (16). The rising number of computers per 100 families reflected the increasingly westernized lifestyles, and more and more divorces triggered stronger mental stress, both of which cancer incidence is attributed to (17, 18). More health expenditure and medical insurance cost are probably attributed to the decline of age-standardized cancer mortality in urban Shanghai, which were consistent with the published results (19, 20). Even though correlations between socioenvironmental factors and cancer incidence and mortality are observed, causal relationships remain unproven. In addition, the impacts of genetic predisposition, diagnostic advancements, aging population effects, and migration effects on cancer burden were not estimated, which probably influence the current results. Therefore, it is necessary for prospective robust studies to validate these causal relations.

During Shanghai’s metropolitanization, the cancer incidence patterns shifted to lung, colorectal, and female breast and male prostate cancers, which were also close to our many efforts on fighting against cancers. Primary prevention actions have been implemented since the 1990s, such as free hepatitis B vaccinations, *Helicobacter pylori* infection screening, and health education regarding cancer risk behavior, causing the decline in hepatitis B virus infection, *Helicobacter pylori* infection, and reflux esophagitis, all cancer risk factors (21, 22). The advanced treatment therapies and increases over time in fruit and vegetable consumption may result in a decline in mortality due to liver, stomach, and esophagus cancers (23). The dramatic increase in thyroid cancer was also observed in other countries, which may reflect “over-diagnosis” through the increasing use of new imaging diagnostic technologies, such as ultrasound and computed tomography (24). Herein thyroid cancer was not listed in the estimation of cancer incidence patterns. Prostate-specific antigen screening may drive the increase in prostate cancer (25). The increase in cervix cancer incidence and mortality may be attributed to the limitations of current HPV screening methods and vaccines (26). The implementation of strict smoking control laws in Shanghai since 1994 may have contributed to stabilizing the rising trend in male incidence (27). The increase in female lung cancer cases is probably due to indoor air pollution caused by cooking and heating using fuel gas and coals, along with soil and water contamination. The implementation of one-child policy since the 1970s and more divorces, along with mammography screening program, may contribute to the increasing trend in female breast cancer incidence. The adoption of westernized lifestyles, characterized by higher obesity rates and physical inactivity, may have an impact on the increase in incidences of breast and colorectal cancers.

The current study have some limitations. Firstly, this study is an ecological study. The trends of cancer burden and socioenvironmental factors were only correlated, resulting in no robust causal relations. Secondly, the distribution of age in cancer was not further explored. Thirdly, cancer data in rural Shanghai from 1973 to 2001 was not obtained.

## Conclusion

The longitudinal cancer data from the real world, which span decades, reported here and Shanghai’s experience in cancer prevention and control can be a reference for government guidelines in preventing population-level cancer incidence during city development.

## Data Availability

Due to the grounds of our ethics approval, data from this study are unable to be shared. Most of the data supporting the conclusions of this study are available in the Cancer Incidence in Five Continents (CI5) series: Cancer Incidence in Five Continents Volumes I to X by IARC (http://ci5.iarc.fr/CI5I-X/Default.aspx).

## Supplementary material

Supplementary Figure 1 Sensitivity analysis of incidence and mortality trends of each cancer type in rural Shanghai, 2002–2017, and in urban Shanghai, 1973–2017. **(A, B)** Estimated annual percentage change (EAPC) of crude and age-standardized cancer incidence and mortality categorized by gender and district in rural 2002 to 2017 and urban 1973 to 2017. **(C, D)** Trends in cervix and gallbladder incidence and mortality standardized by Segi’s world population in 1960, categorized by gender and district in rural 2002 to 2017 and urban 1973 to 2017.

Supplementary Figure 2 Stacked bar plots depict the cancer patterns of deaths by genders in rural, 2002–2017, and urban, 1973–2017.
