# Supplementary figures and images for: Trends in cancer incidence and mortality in the process of metropolitanization of Shanghai, 1973–2017
Source: Front Oncol. 2025 Aug 1;15:1615492. doi: 10.3389/fonc.2025.1615492 (PMC12353708; doi:10.3389/fonc.2025.1615492)

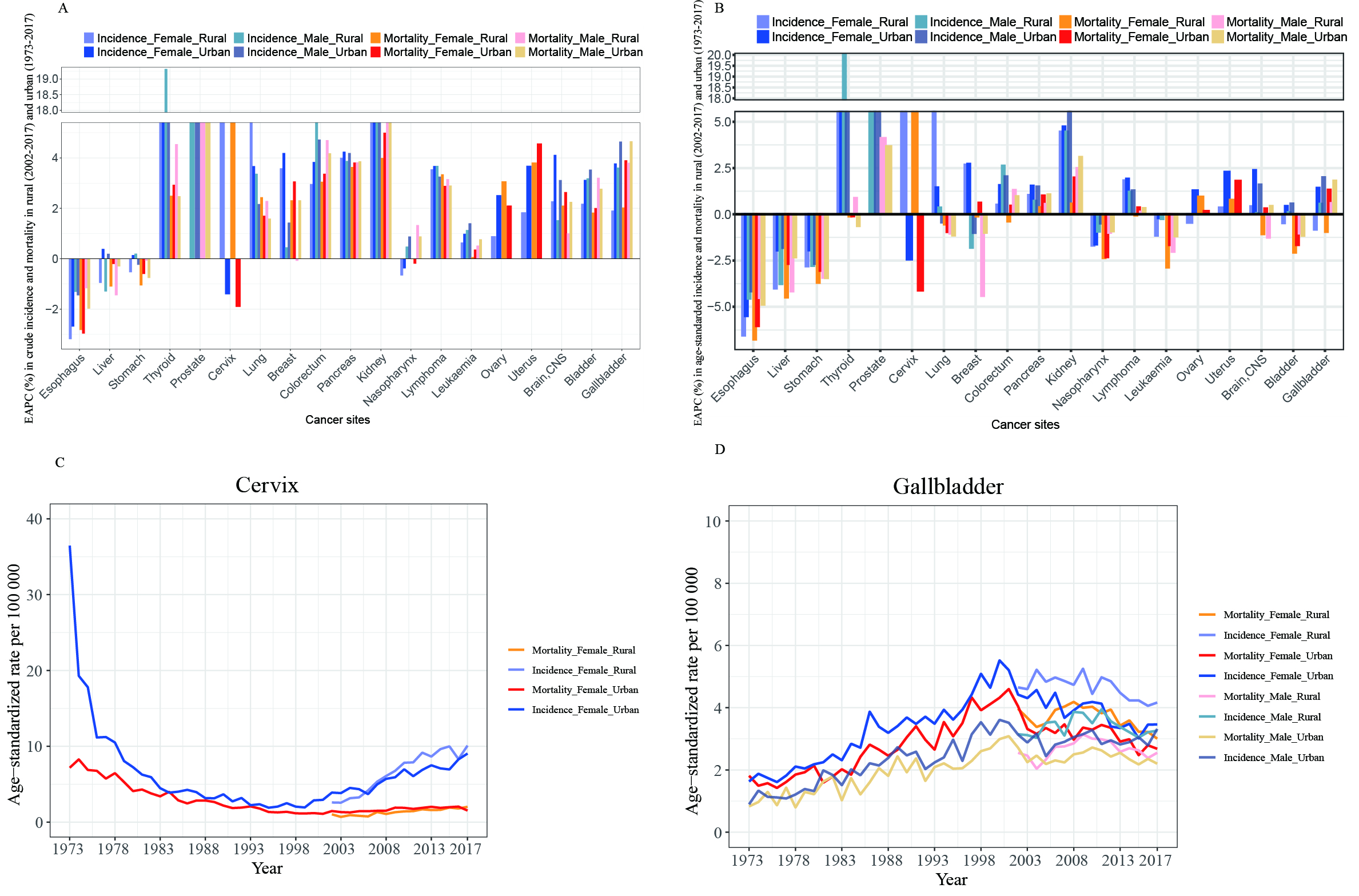

Supplement: Supplementary Figure 1 — Sensitivity analysis of incidence and mortality trends of each cancer type in rural Shanghai, 2002–2017, and in urban Shanghai, 1973–2017. (A, B) Estimated annual percentage change (EAPC) of crude and age-standardized cancer incidence and mortality categorized by gender and district in rural 2002 to 2017 and urban 1973 to 2017. (C, D) Trends in cervix and gallbladder incidence and mortality standardized by Segi’s world population in 1960, categorized by gender and district in rural 2002 to 2017 and urban 1973 to 2017. [file Image1.jpeg]

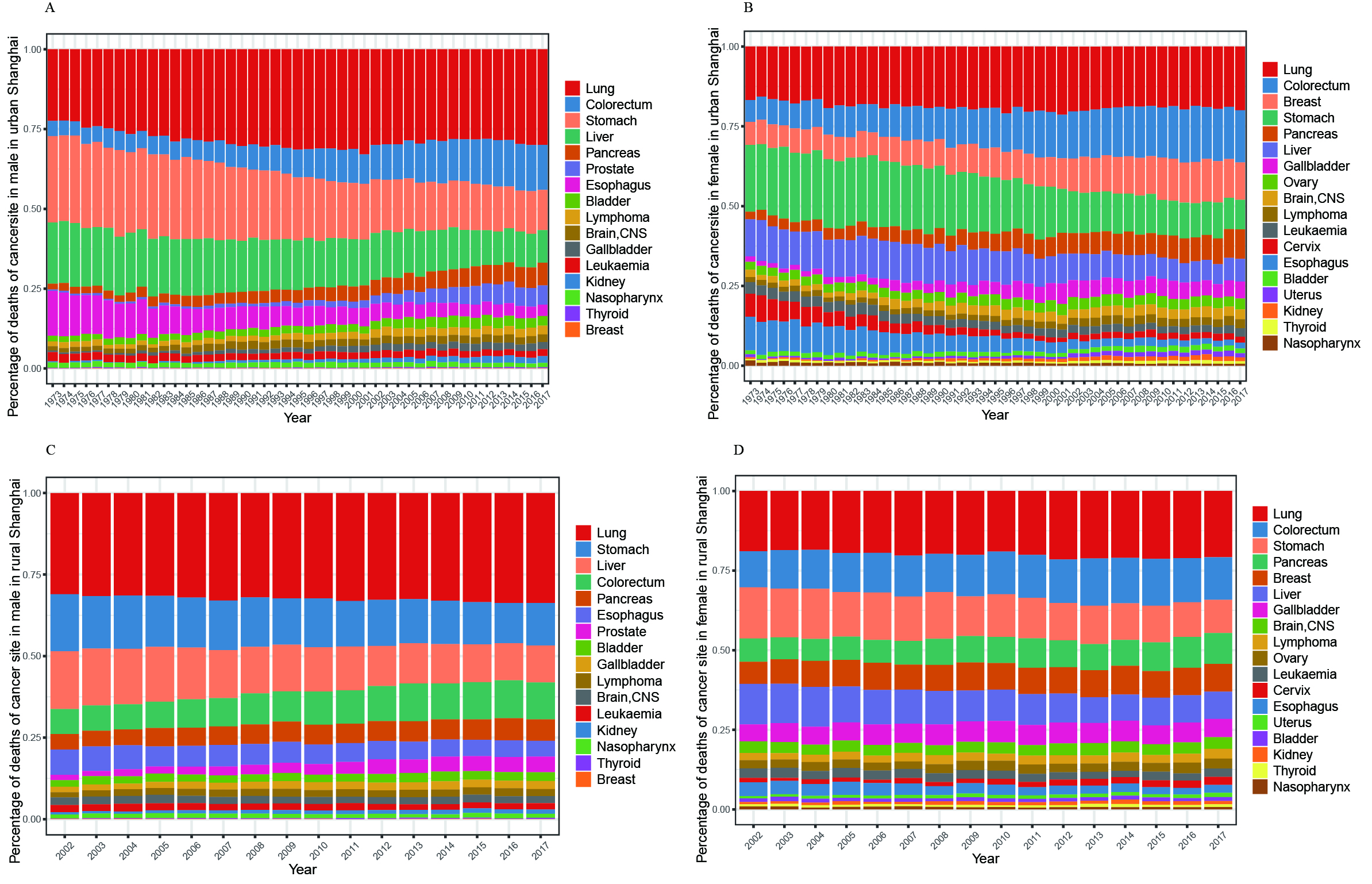

Supplement: Supplementary Figure 2 — Stacked bar plots depict the cancer patterns of deaths by genders in rural, 2002–2017, and urban, 1973–2017. [file Image2.jpeg]
